# Comparative Analysis of Estragole, Methyleugenol, Myristicin, and Elemicin Regarding Micronucleus Formation in V79 Cells

**DOI:** 10.3390/molecules30040806

**Published:** 2025-02-10

**Authors:** Andreas Eisenreich, Lucas Wittek, Marlies Sagmeister, Mia Kruse, Josephine Krüger, Benjamin Sachse, Jakob Menz, Mario E. Götz, Bernd Schäfer

**Affiliations:** Department of Food Safety, German Federal Institute for Risk Assessment, Max-Dohrn-Straße 8-10, 10589 Berlin, Germany

**Keywords:** alkenylbenzenes, genotoxicity, micronucleus assay, viability, apoptosis, necrosis

## Abstract

Alkenylbenzenes occur as natural constituents in a variety of edible plants, in particular those herbs and spices used to give a distinctive flavor to a range of food and feed items. Some alkenylbenzenes with relevance for food, such as estragole and methyleugenol, are known to be genotoxic and carcinogenic in rodents. However, the genotoxic and carcinogenic potential of other structurally related alkenylbenzenes, such as myristicin and elemicin, is still under scientific discussion. Here, we investigated the potential of myristicin and elemicin to induce micronuclei (MN) in V79 cells in comparison to that of estragole and methyleugenol. In addition, we determined the impact of these alkenylbenzenes on cell viability and on the induction of apoptosis and necrosis. All tested alkenylbenzenes affected cell viability in a concentration-dependent manner, albeit to varying degrees. Regarding MN formation, elemicin induced a weak but statistically significant response at 100 µM and 500 µM in the absence of an exogenous metabolizing system (S9 mix). Negative results were obtained for estragole and myristicin at the highest tested non-cytotoxic concentration of 10 µM and 100 µM, respectively. For methyleugenol, the MN assay results were considered equivocal, since the observed change in MN induction was rather small and not supported by a concentration-related trend. These findings indicate that traditional in vitro test systems utilizing exogenous metabolizing systems have limited explanatory power with regard to the genotoxic potential of alkenylbenzenes.

## 1. Introduction

Alkenylbenzenes are secondary plant metabolites, occurring as characteristic flavor constituents of various herbs and spices, such as nutmeg, fennel, and basil [1]. As they are components of essential oils [2], particularly high concentrations can be found in food products made from aromatic parts of different herbs and spices, such as basil-based oily condiments (e.g., “pesto”), non-alcoholic or alcoholic beverages (e.g., fennel tea or anis-based liqueurs), or plant-based food supplements [3].

For many alkenylbenzenes occurring in food, the toxicological data required for a conclusive risk assessment are sparse [1,2]. However, some alkenylbenzenes, such as estragole (EST), methyleugenol (ME), and safrole (SAF), are well studied and known to be genotoxic and carcinogenic in rodents [4,5,6]. Tumors, observed mainly in the liver but also in other organs, seem to be induced by reactive metabolites. These are formed via cytochrome P450 (CYP)-mediated 1′-hydroxylation followed by sulfotransferase (SULT)-catalyzed sulfonation [3]. The resulting allylic sulfate esters spontaneously form electrophilic carbocation intermediates [3]. They are expected to react with cellular nucleophiles, including DNA and proteins, and are therefore likely to act as ultimate carcinogens [4,5,6,7]. Due to their genotoxic potential, the addition of the above-mentioned alkenylbenzenes as such to food has already been prohibited and maximum levels of these substances, when naturally present in flavorings and food ingredients with flavoring properties as well as in certain compound foods, have been defined in the European Union (EU) via Regulation (EC) No 1334/2008.

Based on structural similarities to EST, ME, and SAF, it is reasonable to assume that other allylic alkenylbenzenes, like myristicin (MYR) and elemicin (ELE), may also exhibit a genotoxic and carcinogenic potential [1]. However, as is also true for other structurally related substances, the toxicological profiles of MYR and ELE, including genotoxicity, have not yet been comprehensively studied. Those data, together with reliable data on exposure (which are still lacking), are required for a conclusive risk assessment which could support regulation in the EU [3].

**Figure 1:**
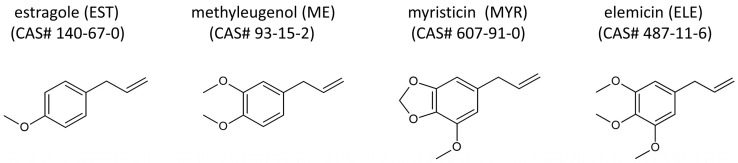
Structural formulas of estragole (EST), methyleugenol (ME), myristicin (MYR), and elemicin (ELE). CAS# = Chemical Abstracts Service number.

In this study, we asked whether a high-throughput micronucleus assay (MNT) with V79 cells can provide relevant comparative insights into the genotoxic potential of yet less characterized alkenylbenzenes. As examples, we analyzed MYR and ELE in comparison to the structurally related and well-studied alkenylbenzenes EST and ME (Figure 1). Moreover, we characterized the differential composition of the cytotoxic effects of these alkenylbenzenes and analyzed the mRNA expression of different exemplary metabolic factors in Chinese hamster (ch) V79 cells. This information may help to ameliorate—at least in part—the insufficient data situation regarding alkenylbenzenes in food.

## 2. Results

### 2.1. Impact of EST, ME, MYR, and ELE on Micronucleus Formation in V79 Cells

**Figure 2:**
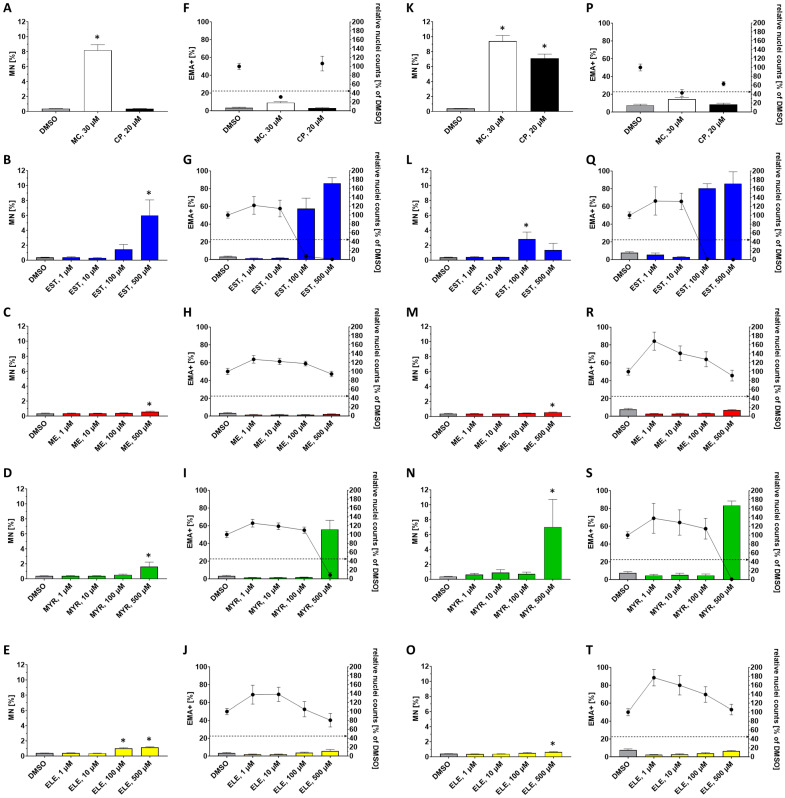
Effect of the studied alkenylbenzenes on micronucleus (MN) formation and viability in V79 cells exposed for 4 h and sampled after 24 h. Shown is the percentage of MN events in the absence (**A**–**E**) or in the presence (**K**–**O**) of exogenous metabolization (S9) as well as the percentage of EMA-positive (EMA+) cells and relative nuclei counts (used as cytotoxicity marker and as indicator of cell viability) related to 1% DMSO (negative control) in the absence (**F**–**J**) or in the presence (**P**–**T**) of S9, respectively. Cells were treated with 1–500 µM estragole (EST), methyleugenol (ME), myristicin (MYR), or elemicin (ELE), respectively. The positive controls were 30 µM mitomycin C (MC; −S9) and 20 µM cyclophosphamide (CP; +S9). In (**F**–**J**,**P**–**T**), the interlinked or single dots represent the relative nuclei counts (% of DMSO) and the dashed line indicates a cytotoxicity limit of 55%. The mean ± SEM of at least 3 independent experiments is shown. (*) = *p* < 0.05.

The influence of EST, ME, MYR, and ELE (1–500 µM each) on micronucleus (MN) formation, the percentage of dead (ethidium monoazide positive; EMA+) cells, and cytotoxicity based on relative nuclei counts was analyzed in V79 cells sampled after 24 h including 4 h exposure in absence (Figure 2A–J), as well as in the presence (Figure 2K–T) of an exogenous metabolizing system (S9).

Positive Controls (−S9): Treatment of V79 cells with 30 µM mitomycin C (MC, used as positive control for an alkylating agent in the absence of S9) significantly increased the frequency of MN. In contrast to MC, 20 µM cyclophosphamide (CP, used as positive control in the presence of S9) had no notable influence on MN generation in the absence of exogenous metabolization (Figure 2A), as expected. Changes in EMA+ cells and relative nuclei counts were considered to fall in an acceptable range (Figure 2F).

EST (−S9): EST did not induce a significant increase in MN frequency up to the highest tested concentration of 10 µM, at which no critical cytotoxicity was observed (Figure 2B). At 100 µM and 500 µM, excessive cytotoxicity was evident in a high percentage of EMA+ cells, as was a sharp decrease in relative nuclei counts (Figure 2G). Therefore, the reliable measurement of MN induction was not possible in this concentration range.

ME (−S9): Treatment of cells with 1–100 µM ME had no effect on MN induction, whereas 500 µM ME induced a small but significant (*p* < 0.05) increase in MN formation (Figure 2C). ME treatments at 1–500 µM had no impact on the percentage of EMA+ cells or relative nuclei counts (Figure 2H). However, since the increase in MN induction at 500 µM was minor and not supported by a concentration-related trend, this result was considered equivocal.

MYR (−S9): Similar to EST, MYR produced excessive cytotoxicity at 500 µM, characterized by an increased rate of EMA+ cells and a sharp decrease in relative nuclei counts. At 1–100 µM, the concentration range at which cytotoxicity did not interfere with MN measurements, no significant difference in MN formation was found (Figure 2I).

ELE (−S9): Treatment of V79 cells with ELE significantly increased the MN formation rate at 100 µM and 500 µM (Figure 2E). At 1–500 µM ELE, no excessive cytotoxicity based on the EMA+ cell rate or relative nuclei counts was observed. However, at 500 µM ELE, a slightly increased amount of EMA+ cells and moderately reduced relative nuclei count, compared to the lower concentrations of ELE, was found (Figure 2J). As the increase in MN was rather small but concentration-related and no excessive cytotoxicity occurred, this was interpreted as a weakly positive result.

Positive Controls (+S9): Both 30 µM MC as well as 20 µM CP significantly increased the occurrence of MN after 24 h (Figure 2K), the latter demonstrating sufficient effectiveness of the exogenous metabolic activation system. Changes in EMA+ cells and relative nuclei counts were considered to fall in an acceptable range (Figure 2P).

EST (+S9): Treatment with 1–10 µM EST had no effect on MN formation, EMA+ cells, and relative nuclei counts (Figure 2L,Q), whereas treatment with 100–500 µM EST produced excessive cytotoxicity. Therefore, also in the presence of S9, a reliable measurement of MN induction was not possible at the two highest concentrations.

ME (+S9): Similar to the results obtained in the absence of S9, 1–100 µM ME had no influence on MN formation, whereas 500 µM ME led to a slight but significant increase of the MN induction rate after 24 h (Figure 2M). There was a slight increase in the EMA+ cell rate at 500 µM and a trend towards a decrease in the relative nuclei count compared to lower concentrations of ME but not to the control (Figure 2R). Since the observed change in MN induction was rather small and not supported by a concentration-related trend, this result was considered equivocal.

MYR (+S9): Treatment of V79 cells with 500 µM MYR was accompanied by excessive cytotoxicity, indicated by a strong increase in EMA+ cells and a sharp decrease in relative nuclei counts, compared to the negative control (Figure 2S). In the concentration range of 1–100 µM (Figure 2S), at which no critical cytotoxicity occurred, no significant increase in MN frequency was observed (Figure 2N).

ELE (+S9): Treatment of cells with ELE slightly but significantly induced the formation of MN at 500 µM (Figure 2O). Similar to ME, there was a slight trend towards an increase in EMA+ cell rate and a decrease in relative nuclei counts compared to lower concentrations of ME but not to the control in this setting (Figure 2T). Since the increase in MN induction at 500 µM was minor and not supported by a concentration-related trend, this result was considered equivocal.

In conclusion, ELE induced a weak increase in MN formation at 100 µM and 500 µM in absence of an exogenous metabolizing system (S9 mix). Negative results were obtained for EST and MYR up to the highest tested concentration of 10 µM and 100 µM, respectively, at which no critical cytotoxicity occurred. For ME, the results of the MN assay were considered equivocal.

### 2.2. Impact of EST, ME, MYR, and ELE on Apoptosis and Necrosis in V79 Cells

The influence of EST, ME, MYR, and ELE exposure on the proportions of V79 cells in viable, early apoptotic, and late apoptotic/necrotic states were assessed after 24 h including 4 h exposure in the absence or in the presence of an exogenous metabolizing system, respectively.

**Figure 3:**
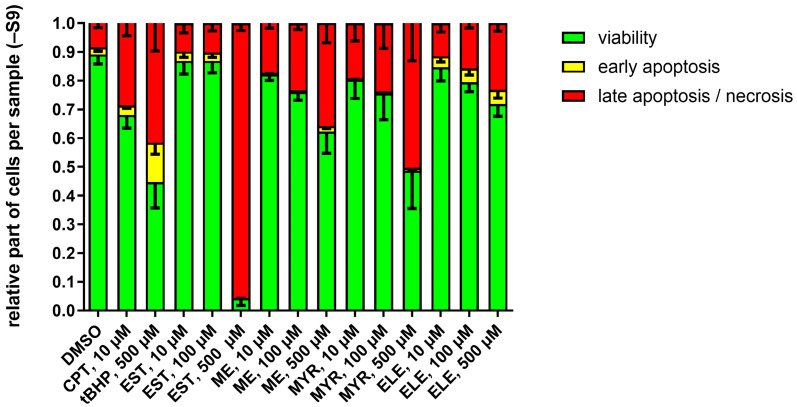
Effect of alkenylbenzenes on the proportions of viable, early apoptotic, and late apoptotic/necrotic cells in the absence of exogenous metabolization (S9). V79 cells were exposed for 4 h and sampled after 24 h. Treatment was performed with 10–500 µM estragole (EST), methyleugenol (ME), myristicin (MYR), or elemicin (ELE), respectively, 10 µM camptothecin (CPT) or 500 µM tert-butylhydroperoxide (tBHP) were used as positive controls, and 1% DMSO was used as negative control. The mean ± SEM of at least 3 independent experiments is shown.

In the absence of metabolic activation (−S9), 10 µM of the positive control camptothecin (CPT) reduced cell viability and increased early- and late-stage apoptosis and necrosis compared to DMSO (Figure 3). The same applied for 500 µM tert-butylhydroperoxide (tBHP), a positive control for peroxide-induced apoptosis and necrosis. Treatment of V79 with 10 µM or 100 µM EST had no notable effect on cell viability or induction of apoptosis and necrosis. However, 500 µM EST obviously lowered cell viability and increased apoptosis and necrosis. Compared to the negative control, 10–500 µM ME, MYR, and ELE led to a dose-dependent and moderate reduction of cell viability and to an increase of early- and late-stage apoptosis and necrosis (Figure 3).

**Figure 4:**
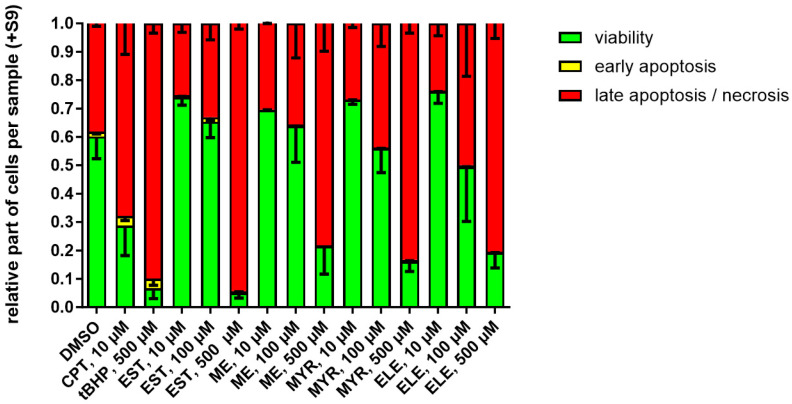
Effect of alkenylbenzenes on the proportions of viable, early apoptotic, and late apoptotic/necrotic cells in the presence of exogenous metabolization (S9). V79 cells were exposed for 4 h and sampled after 24 h. Treatment was performed with 10–500 µM estragole (EST), methyleugenol (ME), myristicin (MYR), and elemicin (ELE), respectively, 1% DMSO was used as negative control, and 10 µM camptothecin (CPT) or 500 µM tert-butylhydroperoxide (tBHP) were used as positive controls. The mean ± SEM of at least 3 independent experiments is shown.

In the presence of metabolic activation (+S9), the DMSO control exhibited a lower rate of viable cells and a higher rate of cells in early- and late-stage apoptosis and necrosis, compared to the absence of S9 (Figure 3). Treatment of cells with 10 µM CPT as well as with 500 µM tBHP (positive controls) in the presence of S9 reduced cell viability and increased apoptosis/necrosis, compared to DMSO (Figure 4). Treatment of V79 cells with 10–500 µM EST, ME, MYR, or ELE led to a concentration-dependent decrease of cell viability and to an increase of cells in early- and late-stage apoptosis and necrosis. The intensity of these effects was comparable between the tested alkenylbenzenes in the presence of S9 (Figure 4).

### 2.3. Expression of Selected Genes Encoding Cytochrome P450 and Sulfotransferase Enzymes on mRNA Level in V79 Cells

**Figure 5:**
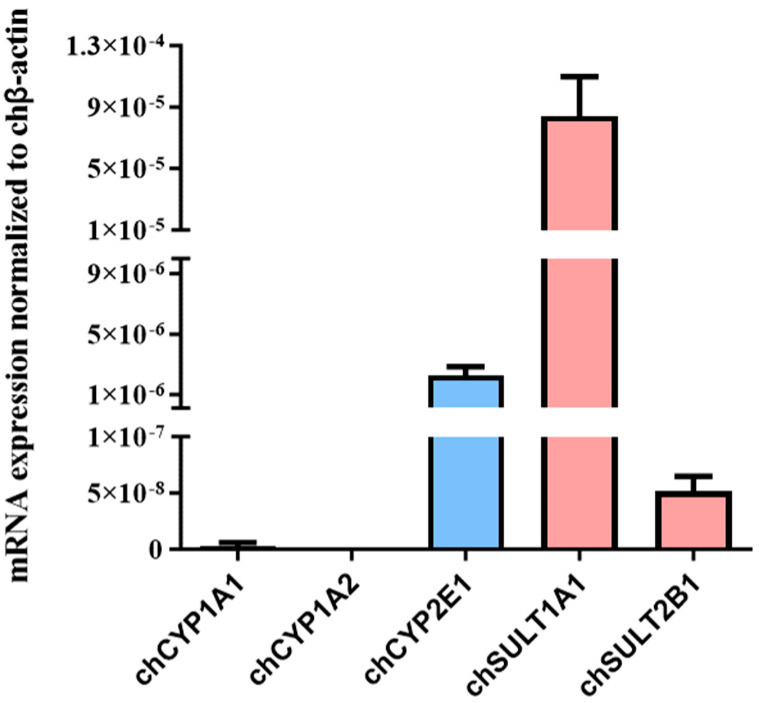
Expression of selected genes encoding cytochrome P450 and sulfotransferase in the V79 cell line. Shown is the mRNA abundance of Chinese hamster cytochrome P450 1A1 (chCYP1A1), 1A2 (chCYP1A2), 2E1 (chCYP2E1), sulfotransferase 1A1 (chSULT1A1), and 2B1 (chSULT2B1) in V79 cells. The abundance of the mRNAs was normalized to that of Chinese hamster beta-actin (chβ-actin) mRNA.

Based on results on functional analyses in V79 cells published in 1990, it is assumed that these cells exhibit negligible endogenous metabolic capacity [8]. However, it is unknown whether these cells express metabolic factors, such as CYPs or SULTs. Therefore, we performed a database search and identified exemplarily representatives of CYPs or SULTs expressed in Chinese hamster (ch) cells. The expression of selected genes encoding chCYP1A1, 1A2, and 2E1, as well as those encoding chSULT1A1 and 2B1, was studied at the mRNA level (Figure 5). In V79 cells, chCYP1A1 and chSULT2B1 mRNA was only detectable at low levels. Compared to chCYP1A1 and chSULT2B1, the expression of chCYP2E1 and chSULT1A1 was much higher, whereas chCYP1A2 was not detectable at the mRNA level (Figure 5).

## 3. Discussion

In recent years, several relevant data gaps were identified regarding alkenylbenzenes in foods, highlighting genotoxicity as one of the most important toxicological aspects [1,9]. To evaluate whether a high-throughput MNT using V79 cells could shed more light on the potential genotoxic effects of less characterized alkenylbenzenes, we performed a comparative analysis using EST, ME, MYR, and ELE as examples.

For the assessment of MN testing results, it is usually recommended to interpret results obtained at cytotoxicity > 55 ± 5% with caution [10]. This is based on the observation that higher levels of cytotoxicity can induce chromosome damage as a secondary effect [11,12]. In this context, Meintieres et al. showed in 2004 that increased apoptosis may also lead to the generation of micronucleated cells, which can potentially affect the outcome of the in vitro MNT, e.g., when performed under extreme conditions [13]. Therefore, in the case of a treatment resulting in excessive cytotoxicity, as observed for higher concentrations of EST and MYR in the present study, increases in MN percentage cannot be interpreted as positive findings.

In the present study, negative MN assay results were obtained for EST at the highest tested concentration of 10 µM, without critical cytotoxicity, while higher concentrations of EST caused excessive cytotoxicity. In line with our findings, EST was also found to be negative in chromosomal aberration tests at 10–1000 µM performed with V79 cells [14,15]. Additionally, predominantly negative in vitro findings regarding the mutagenic potential of EST in the bacterial reverse mutation assay have been published [16,17]. However, this does not exclude that EST induces genotoxicity per se. For example, Martins et al. showed in 2012 that EST induces DNA strand breaks at 250–1000 µM (assessed via an alkaline comet assay) and sister chromatid exchanges (SCE) at 100–750 µM in V79 cells in the absence of S9 [15]. They also found 500–1000 µM EST to induce the formation of DNA adducts in absence of S9 in V79 cells in a dose-dependent manner. Based on their results and the assumption that V79 cells exhibit negligible endogenous metabolic capacity [8], the authors suggested that EST is a weak food-borne genotoxin [15]. Beside this, as comprehensively reviewed elsewhere [18], there is strong evidence of the genotoxic and carcinogenic potential of EST and its metabolites from in vivo studies. Consequently, EST was evaluated as genotoxic and carcinogenic [1,5].

ME is also known to be genotoxic and carcinogenic in rodents [1,4,9]. In our study, however, the in vitro MN assay produced equivocal results for ME. In previous studies, testing of its genotoxic potential in vitro let to negative and positive results, depending on the utilized experimental setting [1,4]. On the one hand, ME was found to be negative in different bacterial test systems, such as the bacterial reverse mutation assay (+/−S9) at 30–300 µg/plate or 0–666 µg/plate and the *Bacillus subtilis* DNA repair test (+/−S9) at 1 mg/disk, as well as in an in vitro chromosome aberration test at 50–500 µg/mL with Chinese hamster ovary (CHO) cells (+/−S9) [17,18]. Moreover, Groh et al. found no significant impact of 10–100 µM ME on the MN frequency in V79 or human colon carcinoma cells (HT29) in the absence of S9 [19,20]. On the other hand, 50–100 µM ME were positive in an unscheduled DNA synthesis (UDS) test in primary cultures of freshly isolated rat hepatocytes [21]. Further, 167 µg/mL ME induced SCE in CHO cells with but not without S9 [18]. ME was also positive in a Comet assay at 10–100 µM with V79 and at 50–100 µM with HT29 cells in absence of S9. Moreover, ME metabolites (methyleugenol-2′,3′-epoxide and 3′-oxomethylisoeugenol) were positive at 10–100 µM in MNT in V79 and at 25–100 µM with HT29 cells in the absence of S9, substantiating the important role of adequate metabolic competence in this context [19,20].

In this study, negative MN assay results were obtained for MYR at the highest tested concentration of 100 µM, at which no critical cytotoxicity occurred, while 500 µM MYR caused excessive cytotoxicity. However, this does not rule out genotoxicity of MYR in other experimental settings, as also discussed for EST. So far, limited data are available on the potential genotoxic effects of MYR. According to a report published by the National Toxicology Program (NTP) in 2019 [22], MYR was assessed to be negative in the bacterial reverse mutation assay at 0–833 µg/plate with or without S9. Moreover, in three-month in vivo studies, no increase in micronucleated polychromatic erythrocytes (PCEs) was found in B6C3F1/N mice. However, 600 mg/kg MYR induced a small but statistically significant increase in micronucleated PCEs in Fisher (F344) rats. MYR was also shown to induce genotoxic effects by Kobets et al. in 2016. They demonstrated in an in vivo avian egg model that 50 mg/egg MYR induced DNA stand breaks in a Comet assay as well as the formation of DNA adducts [23]. Even if our MNT in V79 cells produced negative results, the abovementioned findings of others, especially those obtained in vivo, suggest that MYR exhibits genotoxic potential.

In our experiments, ELE tested positive for MN induction in the absence of S9, whereas an equivocal result was obtained in the presence of S9. This adds to the growing evidence suggesting that ELE has genotoxic potential (reviewed in [1,3]). For example, Hasheminejad et al. [24] showed in 1994 that ELE was positive in an UDS assay (maximum response at 500 µM) performed with rat hepatocytes isolated from male Fischer (F344) rats. Genotoxic effects of ELE were also observed in two in vivo models. In 2016, Kobets and colleagues showed that ELE induced DNA stand breaks in a Comet assay at 20 and 50 mg/egg as well as DNA adduct formation at 50 mg/egg in an avian egg model, indicating genotoxic potential in this setting [23]. More recently, Ishii et al. demonstrated that 400 mg/kg ELE induced genotoxicity in a transgenic rodent model (*gpt* delta rats), as determined by significantly increased *gpt* mutant frequencies [25]. Moreover, they found that 400 mg/kg ELE increased the number and area of glutathione S-transferase placental form-positive foci, which was interpreted as a preneoplastic effect pointing to hepatocarcinogenic potential. Taken together, the aforementioned findings increasingly indicate that ELE exhibits genotoxic potential.

The data obtained from the MNT in V79 cells exposed for 4 h and sampled after 24 h indicate that EST, ME, MYR, and ELE differentially affected relative nuclei counts (as an indicator of cytotoxicity) and rates of dead (EMA+) cells (Figure 2). Therefore, we performed additional experiments to further characterize the impact of EST, ME, MYR, and ELE on cell viability as well as on rates of early- and late-stage apoptosis and necrosis (Figure 3 and Figure 4). Our data show that in V79 cells exposed for 4 h and sampled after 24 h, the fraction of cells in the early phase of apoptosis is in all samples much smaller than that of cells in the late apoptosis/necrosis phase after 24 h. At this relatively late sampling timepoint, the early phase of apoptosis is already over and most of the remaining cells are either viable or in the late apoptosis phase or in necrosis, respectively.

We further found that treatment of V79 cells with 10–500 µM EST, ME, MYR, or ELE, respectively, led to a concentration-dependent decrease of cell viability and increased rate of apoptosis/necrosis after 24 h. As also found in the MNT (Figure 2), the cytotoxic effects of EST and MYR, especially at high concentrations (500 µM), were much more intensive than those of ME and ELE (Figure 3 and Figure 4). In line with our findings, other groups also showed that alkenylbenzenes, such es EST, MYR, eugenol, and *trans*-anethole, reduced cell viability and induced apoptosis in a concentration-dependent manner in different experimental settings [26,27,28,29]. For example, Martins et al. demonstrated in 2011 MYR to reduce cell viability and to induce apoptotic processes in DNA-repair-deficient EM9 Chinese hamster ovary cells, especially at higher doses > 500 µM [27]. In another study published in 2015, Andrade and colleagues showed that 25 µg/mL EST induced cytotoxic effects in human MCF-7 and NCIH292 cells after 24 h [26].

Moreover, we found that the effects of EST, ME, MYR, or ELE, as well as the positive controls (CPT and tBHP), on viability and apoptosis/necrosis were much higher in the presence of S9, compared to experiments performed in the absence of S9. This is in line with findings of others also showing increased cytotoxic effects of different metabolically activated substances, such as eugenol or benzo[a]pyrene, in V79 cells in the presence of S9 [30,31]. Further substantiating the cytotoxic effects of S9, Cox et al. demonstrated in 2015 that higher concentrations of human S9 also led to higher cytotoxicity in different settings and cell lines, including Chinese hamster cells [32].

Overall, the results of our study, as well as data reported in the literature, clearly show that alkenylbenzenes often give inconclusive or conflicting results in in vitro genotoxicity studies, whereas many derivatives have been shown to be genotoxic in vivo. These conflicting results are probably due to the complex bioactivation of alkenylbenzenes leading to genotoxic metabolites. The main pathway leading to the bioactivation of alkenylbenzenes is thought to be side-chain hydroxylation followed by sulfo conjugation to give reactive sulfate esters [1]. However, when external metabolizing systems such as S9 are used, these molecules are formed outside the cells. As the sulfate esters are charged, these metabolites are often unable to penetrate the cell sufficiently. As a consequence, such an approach may lead to cytotoxicity rather than genotoxicity, as has been demonstrated in the Ames test for some furan derivatives that are also activated via the SULT pathway [33]. This may also explain, in part, the higher cytotoxicity that has been observed in our study in the presence of S9. Thus, equivocal or weakly positive in vitro results, as observed in our study for ME and ELE and in some other studies reported in the literature for these and other alkenylbenzenes, may be a result of different factors, e.g., the cell line exhibiting residual metabolic activity (such as indicated by mRNA expression of different CYPs and SULTS in the present study), the formation of other genotoxic metabolites, or artefacts, e.g., those caused by cytotoxicity. However, these results may not appropriately reflect the SULT-mediated bioactivation of alkenylbenzenes that is thought to be of high toxicological relevance.

Thus, at this time, standard in vitro genotoxicity tests, including the MNT applied in our study, are currently not suitable models to investigate the genotoxic potency of alkenylbenzenes. The same is probably true for other compounds that are bioactivated to charged metabolites. In this context, mutagenicity analyses in vitro, e.g., via the *hypoxanthine phosphoribosyl transferase* (*HPRT*) gene test could—at least in part—help to approach this issue.

However, in vitro assays to investigate the mutagenic and genotoxic potency of alkenylbenzenes need to be performed with cell lines that express functional phase I (activation) and phase II (conjugation) metabolic enzymes [34,35]. In the case of alkenylbenzenes, at least functional CYPs, SULTs, and glutathione transferases as can be found in the liver would be needed to mimic in vivo metabolism by in vitro test systems [1,36]. This may be reached by using cell lines able or induced to proliferate and to express these enzymes (e.g., cell division as well as metabolically competent liver cells or artificial genetically modified V79 cells expressing specific CYPs and SULTs) in the in vitro MNT or HPRT gene mutation test [34,37,38,39,40]. In addition, the presence of sufficient amounts of co-factors required for the metabolic activation of alkenylbenzenes must be ensured [3]. As for now, in vivo models, in particular the transgenic rodent assay, appear to be the most appropriate test systems to address the mutagenic and genotoxic potential of this class of compounds. Results obtained from in vivo tests could also be used to get an impression of the relative potency of individual alkenylbenzenes [41].

On the other hand, animal testing is increasingly being avoided due to ethical obligations and high costs. In fact, the genotoxicity testing strategy of the European Food Safety Authority (EFSA) only recommends a battery of Ames tests and MNT as a first step. If the results of this battery are negative, no in vivo follow-up is recommended [42]. In turn, the genotoxic activity of alkenylbenzenes would not be detected by the standard procedure.

## 4. Materials and Methods

### 4.1. Cell Culture

In this study, we used V79 cells (Chinese hamster lung fibroblasts) for assessment of genotoxic effects induced by EST, ME, MYR, and ELE. These cells offer different features useful for adequate performance of the MNT. V79 have a doubling time of about 12 h, which is helpful, since cell division is essential for the formation and detection of MN [34,43]. Moreover, these cells exhibit a high sensitivity for genotoxins, due to a reduced DNA repair capacity [44]. V79 cells were cultured in Dulbecco’s Modified Eagle Medium (DMEM; Pan-Biotech, Aidenbach, Germany) containing 10% fetal bovine serum (FBS; Pan-Biotech, Aidenbach, Germany) and 1% penicillin/streptomycin (Capricorn Scientific, Ebsdorfergrund, Germany) at 37 °C in a humidified incubator (5% CO_2_, 95% air).

For experimental testing, V79 cells were treated with 1, 10, 100, and 500 µM EST (Chemical Abstracts Service number (CAS#) 140-67-0, purity = 98%), ME (CAS# 93-15-2, purity > 98%), MYR (CAS# 607-91-0, purity ≥ 85%), and ELE (CAS# 487-11-6, purity = 98%), each provided by Sigma-Aldrich, Taufkirchen, Germany. The selection of the dose range used for alkenylbenzene treatment was based on results of preliminary apoptosis/necrosis tests performed as described below. Treatment was performed for 4 h in the presence (+) or the absence (−) of a 10% S9 mix consisting of 2.85 mL liver S9 (supernatant fraction from homogenized liver after centrifugation at 9000× *g*) from 5- to 6-week-old male Sprague Dawley rats induced with phenobarbital and 5,6-benzoflavone (Trinova Biochem GmbH, Gießen, Germany), 4.65 mL sterile water, and 15 mL co-factor solution (8.0 mM MgCl_2_/6H_2_O (Supelco, St. Louis, MO, USA), 33.0 mM KCl (Merck, Darmstadt, Germany), 67.2 mM Na_2_HPO_4_/2H_2_O (Merck), 15.8 mM NaH_2_PO_4_/2H_2_O (Merck), 5.62 mM glucose-6-phosphate (Carl Roth, Karlsruhe, Germany), 4.1 mM NADPH (Carl Roth), and 4.3 mM NADH (Carl Roth)). This was done in a volume of 500 µL/well (+S9: 350 µL DMEM + 100 µL S9 mix + 50 µL DMEM with substance or control; −S9: 450 µL DMEM + 50 µL DMEM with substance). After that, the medium was replaced by fresh medium for a further 20 h (recovery phase). As controls for genotoxicity, cells were treated with 20 µM cyclophosphamide (CP, positive control in the presence of S9; Sigma-Aldrich) or with 30 µM mitomycin C (Mito, positive control in the absence of S9; Sigma-Aldrich). All test substances and positive controls were solved in dimethyl sulfoxide (DMSO; Merck) and 1% DMSO was used as negative control.

### 4.2. Micronucleus Test

For the treatment phase, 8000 V79 cells/well were seeded in 48-well plates for 24 h in 500 µL DMEM. After that, medium was changed and cells were treated as described above. After 24 h, medium was removed and the percentages of MN and dead (EMA+) cells, as well as cytotoxicity based on relative nuclei counts (as % of DMSO control) were determined via flow cytometry using the MicroFlow In Vitro-250/50 Kit (NC1938779, Litron Laboratories, Rochester, NY, USA) following the manufacturer’s protocol. Flow cytometric analyses were done using the BD Accuri™ C6 Plus (Biosciences Products, Becton, NJ, USA) device.

### 4.3. Apoptosis/Necrosis Assay

At 24 h prior to treatment, 8000 V79 cells/well were seeded in DMEM in 48-well plates. Next, the medium was changed and V79 cells were treated with 10, 100, and 500 µM EST, ME, MYR, ELE, 1% DMSO (negative control), and 10 µM CPT (Sigma-Aldrich) or 500 µM tBHP (Sigma-Aldrich), respectively, as positive controls (+/−S9). Treatment was done as described above. Post 24 h, the supernatant was transferred to a 2 mL tube and kept on ice. Then, cells were washed with 500 µL PBS/well which was then conveyed to a 2 mL tube. Next, V79 cells were detached with 100 µL trypsin/well for 2–3 min and 500 µL DMEM/well as the stop reagent. Then, cells solved in 600 µL trypsin/DMEM were pooled with the treatment medium and PBS in the corresponding 2 mL tube. All samples were centrifugated for 10 min at 300× *g* and 4 °C (Multifuge 3SR+, Thermo Fisher Scientific, Waltham, MA, USA) and the supernatant was discarded. Cells were washed with 100 µL calcium buffer (0.1 M HEPES, pH 7.4; 1.4 M NaCl; 25 mM CaCl_2_; each provided by Carl Roth), centrifugated (10 min at 300× *g* and 4 °C), and the supernatant was discarded. Then, cells were dyed with 80 µL of 5% 7-amino-actinomycin D (7-AAD; BioGems, Via Colinas, CA, USA)/5% Annexin-V-phycoerythrin (AV-PE; Biosciences Products)/90% calcium buffer mix per sample for 20 min in the dark on ice. The measurement was done via flow cytometry using the BD Accuri™ C6 Plus (Biosciences Products) device and following the manufacturer’s protocol. Characterizations of the cell populations were done as follows: PE^−^/7-AAD^−^: viable; PE^+^/7-AAD^−^: early-apoptotic; and PE^−/+^/7-AAD^+^: late-apoptotic and necrotic.

### 4.4. Quantitative Real-Time Polymerase Chain Reaction

Prior to the performance of quantitative real-time polymerase chain reaction (qRT-PCR), total RNA was isolated from V79 cell using the RNeasy mini Kit (Qiagen, Hilden, Germany) following the manufacturer’s instructions, 1 μg of total RNA was reverse transcribed using the High-Capacity cDNA Reverse Transcription Kit (Thermo Fisher Scientific), And 1 µL complementary (c)DNA and 9 µL master mix (2,8 µL water; 5 µL Absolute qPCR SYBR Green ROX mix and 1.2 µL primer (5 µM), respectively) were mixed per well. For qRT-PCR, an AriaDX (Agilent technologies, Waldbronn, Germany) was used. Conditions were 95 °C, 15 s; 40 cycles: 95 °C, 15 s; and 60 °C, 1 min; followed by (each 1x) 60 °C, 10 min; 95 °C, 15 min; 60 °C, 20 min; and 95 °C, 15 min). Determined was the expression of Chinese hamster *(ch)CYP1A1* (all 5′->3′; forward: AGCTTCTGCATCCTCTTGCT; reverse: AAGGGTCAAAGTGGCCAACC), *chCYP1A2* (forward: TAGCCTCAGACCCAACCTCA; reverse: TCTGCCGTTAGCTTCTGCAA), *chCYP2E1* (forward: CGGGATGTGACGGACTGTTT; reverse: AGTCCGCCAAAGTCACAGAA), *chSULT1A1* (forward: GGGTGCCCTTCCTTGAGTTT; reverse: GCAAGGACAGAGGCAGATGT), chSULT2B1 (forward: GGGTTCATGCACACTCTGGA; reverse: CACGTGGTACCTGACTTGGG), normalized against the expression of actin (*chActin:* forward: CACCACAGCTGAGAGGGAAA; reverse: GGAGGAAGAGGATGCAGCAG) in V79. 

### 4.5. Statistical Analysis

All data were expressed as mean ± SEM. Data were analyzed by Student’s *t*-test or one-way ANOVA as appropriate. A probability value (*p*) < 0.05 was regarded as significant. Statistical analyses were done using GraphPad Prism, version 10.1.2 (GraphPad Software, Boston, MA, USA).

## 5. Conclusions

In summary, the findings reported and discussed in this manuscript emphasize the need to further develop existing in vitro genotoxicity testing systems that do not rely on exogenous metabolizing systems in order to increase the sensitivity to genotoxins that are bioactivated to highly reactive but charged compounds.

## Data Availability

The data presented in this study are available on request from the corresponding author (the data are not publicly available due to privacy).
